# Increasing the permeability of *Escherichia coli* using MAC13243

**DOI:** 10.1038/s41598-017-17772-6

**Published:** 2017-12-15

**Authors:** Claudio Muheim, Hansjörg Götzke, Anna U. Eriksson, Stina Lindberg, Ida Lauritsen, Morten H. H. Nørholm, Daniel O. Daley

**Affiliations:** 1 Department of Biochemistry and Biophysics Stockholm University, Stockholm, Sweden; 2 Chemical Biology Consortium Sweden, Laboratories for Chemical Biology, Umeå University, Umeå, Sweden; 3 Novo Nordisk Foundation Center for Biosustainability, Technical University of Denmark, Kgs., Lyngby, Denmark

## Abstract

The outer membrane of gram-negative bacteria is a permeability barrier that prevents the efficient uptake of molecules with large scaffolds. As a consequence, a number of antibiotic classes are ineffective against gram-negative strains. Herein we carried out a high throughput screen for small molecules that make the outer membrane of *Escherichia coli* more permeable. We identified MAC13243, an inhibitor of the periplasmic chaperone LolA that traffics lipoproteins from the inner to the outer membrane. We observed that cells were (1) more permeable to the fluorescent probe 1-*N*-phenylnapthylamine, and (2) more susceptible to large-scaffold antibiotics when sub-inhibitory concentrations of MAC13243 were used. To exclude the possibility that the permeability was caused by an off-target effect, we genetically reconstructed the MAC13243-phenotype by depleting LolA levels using the CRISPRi system.

## Introduction

**Figure 1:**
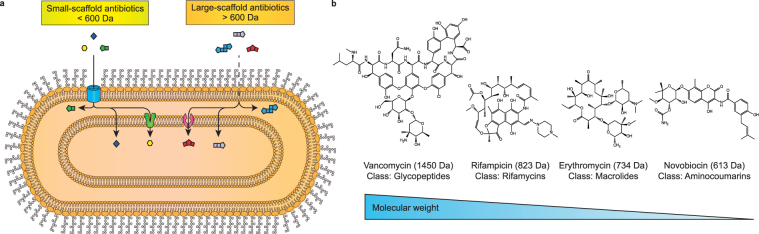
Schematic illustration of antibiotic uptake in *E*. *coli*. (**a**) Antibiotics less than 600 Da (herein called small-scaffold antibiotics) can generally permeate through non-specific outer membrane porins and gain access to the periplasm. Antibiotics larger than 600 Da (herein called large-scaffold antibiotics) exceed the size exclusion limit of outer membrane porins. These antibiotics can presumably diffuse through the outer membrane but the process is inefficient. Once in the periplasm both small- and large-scaffold antibiotics can, in principle, diffuse across the inner membrane or can be inadvertently taken up by membrane embedded transporters. (**b**) Chemical structures of four large-scaffold antibiotics from different classes.

A distinguishing feature of gram-negative bacteria is their outer membrane, which under normal physiological conditions is an asymmetric bilayer that contains mainly lipopolysaccharides (LPS) in the outer leaflet and phospholipids in the inner leaflet^1–3^. This membrane is essentially an impermeable barrier that separates and protects the cell from the extra-cellular *mileu*. ß-barrel porins embedded in the outer membrane maintain cellular homeostasis by selecting the chemistry that enters and leaves the cell. It is generally accepted that ß-barrel porins allow, by passive diffusion, the passage of hydrophilic molecules that are less than 600 Da^4–6^. Molecular shape, flexibility and the presence of an ionisable nitrogen group are also important for diffusion through porins^7^. These exclusion limits enable the uptake of essential nutrients and ions, as well as those antibiotics with small molecular scaffolds. Antibiotics with larger and less favourable scaffolds cannot effectively cross the outer membrane (Fig. 1a). These large-scaffold antibiotics can, in some instances, diffuse through the LPS layer, but the process is inefficient and they are therefore considered ineffective against gram-negative bacteria^8–10^. Examples of large-scaffold antibiotics from four different antibiotic classes are shown in Fig. 1b.

Large-scaffold antibiotics are taken up more efficiently when the biogenesis of the cell envelope is perturbed. For example, in strains with genetic mutations that affect protein trafficking and folding in the cell envelope, or lipopolysaccharide biosynthesis^11–14^. A number of small molecules that inhibit the biogenesis of the cell envelope have been discovered (see^15–24^ for examples). But to our knowledge, only a few of these have been shown to potentiate large-scaffold antibiotics: (1) polymyxins are cationic polypeptide antibiotics that disrupt the LPS layer^15,24,25^, (2) loperamide is an anti-diarrhoeal drug (target unknown) that dissipates the inner membrane potential^17^, (3) A22 is an inhibitor of the actin homologue MreB^23^. In this study we set out to discover new lead molecules that could be used to make the outer membrane of gram-negative bacteria more permeable to large-scaffold antibiotics.

## Results

### A high throughput screen for molecules that make *E. coli* susceptible to vancomycin

**Figure 2:**
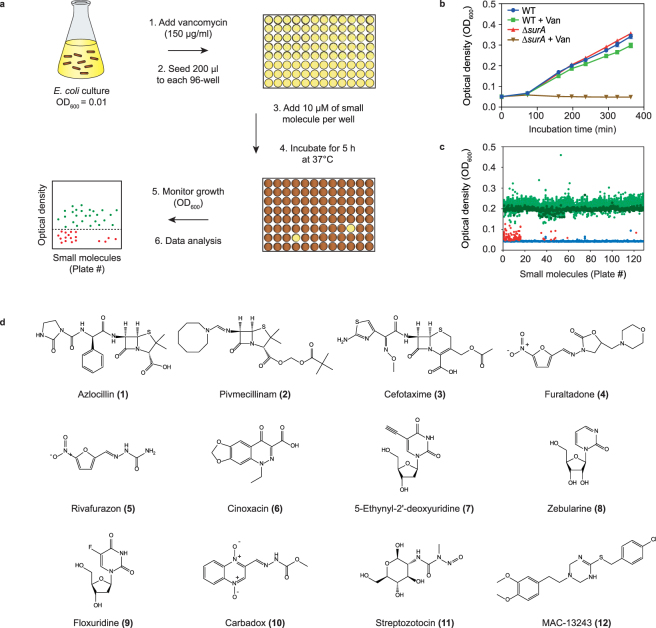
A high-throughput screen to identify small molecules that make *E*. *coli* more permeable. (**a**) An aliquot of *E*. *coli* MC4100 was seeded into individual wells of a 96-well microtiter plate in the presence of a sub MIC of vancomycin and small molecules. The plates were incubated for 5 h at 37 °C and cell growth (OD600) was compared to a DMSO control. (**b**) Growth curves of WT strain and a strain lacking the periplasmic chaperone SurA (Δ*surA*) in the presence or absence of 150 μg mL^−1^ vancomycin (~1/3 MIC). The experiment indicates that growth of the WT strain is unaffected by a sub-lethal concentration of vancomycin, but growth of the Δ*surA* is severely compromised. (**c**) Optical density readings of WT cells grown in the presence of 150 μg mL^−1^ vancomycin (~1/3 MIC) and 10 μM of each small molecule from the LCBKI library. 124 small molecules (red dots) inhibited growth more than 30% compared to the growth control (dark green dots). Sterility controls are illustrated by blue dots and small molecules that did not inhibit cell growth by more than 30% are illustrated as light green dots. (**d**) Chemical structures of 12 small molecules that inhibited cell growth in a vancomycin- and dose-dependent manner. **1–6** were from antibiotic classes that were previously known to function synergistically with vancomycin in gram-negative bacteria, and this category served as positive controls for the screen. **7–12** were not previously known to work in combination with vancomycin and they were chosen for follow up experiments.

We initially carried out a high throughput screen to identify small molecules that make *Escherichia coli* more susceptible to vancomycin. Vancomycin is a glycopeptide antibiotic that exceeds the exclusion limit of ß-barrel porins and therefore cannot pass the outer membrane. However *E. coli* is susceptible to vancomycin when proteins involved in cell envelope biogenesis are inhibited; For example, a strain lacking the periplasmic chaperone SurA has a compromised outer membrane and is susceptible to much lower concentrations of vancomycin than a wild type (WT) strain^12^. Our screen was performed by monitoring the growth of *E*. *coli* MC4100 in a 96-well format, in the presence of a sub-lethal concentration of vancomycin and 28,000 small molecules. We reasoned that if a small molecule increased the permeability of the outer membrane, vancomycin would gain access to the periplasm and cell growth would be inhibited (Fig. 2a). The screening conditions we selected were deemed to be robust as we observed a visible growth difference when the WT MC4100 and the vancomycin sensitive ∆*surA* strain were incubated in the presence of vancomycin at 1/3 the Minimal Inhibitory Concentration (MIC) (Z-score of 0.89; Fig. 2b). The primary screen was initially carried out with 17,500 small molecules from a diverse set of compounds (ChemBridge) at 12.5 µM and in the presence of 100 µg mL^−1^ of vancomycin (1/5 MIC). As this did not yield any significant hits (data not shown) a second screen was carried out. In the second screen 10,500 small molecules were screened from the CBCS primary screening set at 10 µM in presence of 150 µg mL^−1^ of vancomycin (1/3 MIC). This second screen indicated that 124 small molecules inhibited the growth of MC4100 by more than 30% relative to a DMSO control (red data points, Fig. 2c). Roughly half of these molecules exhibited antimicrobial activity in the absence of vancomycin and they were not considered further. Only 12 of the 124 small molecules inhibited cell growth in a vancomycin- and dose-dependent manner (Supplementary Figure 1). Six of these molecules were from antibiotic classes that were previously known to function synergistically with vancomycin in gram-negative bacteria^23,26–28^, and this category served as positive controls for the screen (**1–6** Fig. 2d). The other six molecules were not previously known to work in combination with vancomycin and they were chosen for follow up experiments (**7–12** Fig. 2d). This group included three nucleoside analogues (5-ethynyl-2′-deoxyuridine, zebularine, floxuridine), two molecules that were annotated as antibiotics (carbadox, streptozotocin) and an inhibitor of the periplasmic chaperone LolA (MAC13243)^7^.

### MAC13243 permeabilises the outer membrane of *E. coli*

**Figure 3:**
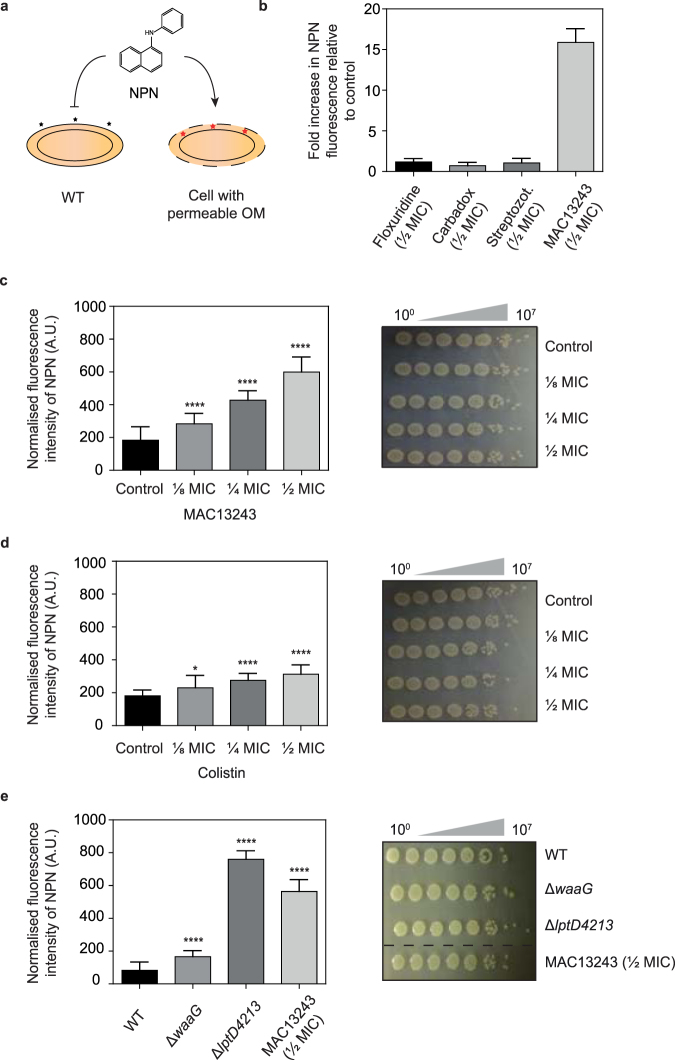
A sub-lethal concentration of MAC13243 makes the outer membrane of *E*. *coli* more permeable. (**a**) The NPN dye can be used to monitor the integrity of the outer membrane. NPN is excluded from WT cells but penetrates into cells with a compromised outer membrane where it binds to the phospholipid layer, resulting in prominent fluorescence. (**b**) *E*. *coli* MC4100 grown in M9 media were exposed to different small molecules (½ MIC), and the permeability of the outer membrane was assessed by measuring the fluorescence of NPN. MICs were determined to be 1 μg mL^−1^ for carbadox, 256 μg mL^−1^ for streptozotocin, 0.002 μg mL^−1^ for floxuridine and 256 μg mL^−1^ for MAC13243. Fluorescence values were compared to cells treated with a solvent control. Note that we did not test all small molecules in the NPN uptake assay, but focussed on those that were readily available and that were representative of a class. For example, floxuridine (**9**) was deemed to be representative of the nucleoside analogues (**7**, **8**). (**c**) *E*. *coli* MC4100 were grown in M9 media then exposed to different concentrations of MAC13234 (MIC = 256 μg mL^−1^) and NPN uptake was monitored (left panel). The increase in fluorescence was deemed to be due to increased permeability of the outer membrane, not cell lysis, since the amount of MAC13243 used did not affect cell viability (right panel). In these experiments cell aliquots were harvested after the NPN uptake assays, 10-fold serially diluted and spotted on LB agar. All data (mean ± SD) are from four experiments. ****p < 0.0001 (unpaired t-test.). (**d**) As for panel c except that *E*. *coli* MC4100 were exposed to different concentrations of colistin (MIC = 1 μg mL^−1^). (**e**) As for panel c except that the permeability of different *E*. *coli* strains was assessed.

We used the fluorescent probe 1-*N*-phenylnapthylamine (NPN) to determine if the molecules identified in the screen were causing the outer membrane of *E*. *coli* to be more permeable. NPN is a small molecule (219 Da) that cannot effectively cross the outer membrane. It is weakly fluorescent in aqueous solution but fluoresces strongly when it binds to phospholipids. This property can be exploited to probe the permeability of the outer membrane^29,30^: WT cells are weakly fluorescent since NPN is not effectively taken up, but strains with a compromised outer membrane are fluorescent since NPN can access the periplasmic space and the phospholipids of the inner and outer membranes (Fig. 3a). When we carried out NPN uptake assays in cells treated with a sub-lethal concentration of floxuridine, carbadox, streptozotocin and MAC13243, we observed that only cells treated with MAC13243 became fluorescent (Fig. 3b). We did not test 5-ethynyl-2′-deoxyuridine and zebularine since they were deemed to be analogues of floxuridine (Fig. 2d). The amount of fluorescence observed in cells treated with MAC13243 was 15x higher than the DMSO control (Fig. 3b) and was concentration dependent (Fig. 3c). To gauge how permeable the cells treated with MAC13243 had become, we compared the NPN fluorescence values to those seen in cells treated with a sub-inhibitory concentration of colistin, a polymyxin antibiotic that disrupts the LPS layer^15,24^. We observed that MAC13243-treated cells were more permeable than colistin-treated cells (Fig. 3c vs d). We also compared the NPN fluorescence values of MAC13243-treated cells to strains that were known to be more permeable. This comparison included a deep-rough strain that lacked a glycosyltransferase required for LPS synthesis (∆*waaG*)^12,31^ and a strain carrying a deletion in a protein involved in LPS trafficking (*lptD4213*)^32^. These data indicated that cells treated with a sub-lethal concentration of MAC13243 were more permeable to NPN than the ∆*waaG* strain, and less permeable than the *lptD4213* strain (Fig. 3e). Taken together these data indicate that a sub-lethal concentration of MAC13243 causes the outer membrane of *E*. *coli* to be more permeable to NPN. The molecular reason why compounds **7**–**11** made cells more susceptible to vancomycin in the original screen remains to be determined.

### MAC13243 sensitises *E. coli* to large-scaffold antibiotics

**Figure 4:**
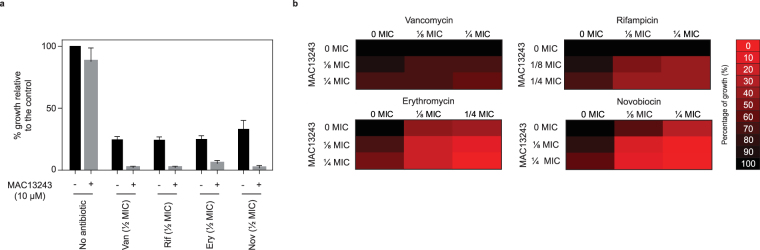
MAC13243 makes *E*. *coli* more susceptible to some large-scaffold antibiotics. (**a**) *E*. *coli* MC4100 cells were exposed to 10 μM MAC13234, or large-scaffold antibiotics (½ MIC), or both. Cells were grown in LB for 18 h at 37 °C and cell growth was determined by measuring the optical density (OD_600_). MICs were determined to be 512 μg mL^−1^ for vancomycin, 16 μg mL^−1^ for rifampicin, 256 μg mL^−1^ for erythromycin and 64 μg mL^−1^ for novobiocin. (**b**) MAC13243 functions synergistically with some large-scaffold antibiotics. Heat plots showing growth inhibition of *E*. *coli* MC4100 in the presence of MAC13243 and vancomycin, rifampicin, erythromycin or novobiocin (in M9 media). Percentage of growth is illustrated with different colours where black represents 100% growth and red 0% growth. These data were used to calculate FICIs (see text for details). MICs were determined to be 256 μg mL^−1^ for MAC13243, 128 μg mL^−1^ for vancomycin, 8 μg mL^−1^ for rifampicin, 256 μg mL^−1^ for erythromycin and 1024 μg mL^−1^ for novobiocin.

To determine if the permeability observed in MAC13243-treated cells could be exploited to increase the uptake of large-scaffold antibiotics, we monitored cell growth in the presence of sub-lethal concentrations of vancomycin, rifampicin, erythromycin or novobiocin (Fig. 1b). In these experiments the assay conditions used were similar to those used in the original screen, except that antibiotics were used at ½ MIC. We observed that growth of MC4100 was only slightly perturbed when 10 µM MAC13243 was present, moderately perturbed when the large-scaffold antibiotics were present at ½ MIC, and severely perturbed when both MAC13243 and large-scaffold antibiotics were present (Fig. 4a). The most likely explanation for these data is that 10 µM MAC13243 increased the permeability of the outer membrane and allowed the large-scaffold antibiotics to enter more efficiently.

To quantify how efficiently MAC13243 was working with the large-scaffold antibiotics we carried out chequerboard assays, which are a commonly used methodology to determine if two drugs work synergistically. In the chequerboard assays the assay conditions were slightly different (see methods) and growth inhibition was evaluated in the presence of serial dilutions of MAC13243 and the large-scaffold antibiotics. The data were then used to calculate a Fractional Inhibitory Concentration Index (FICI). Following community guidelines the combinations were deemed to work synergistically if the FICI was ≤ 0.5^33^. Using this mathematical assessment we observed that MAC13243 worked synergistically with erythromycin and novobiocin but not with vancomycin or rifampicin (Fig. 4b). MAC13243 did not work synergistically with the large-scaffold antibiotics when we carried out chequerboard assays with two clinical isolates, *E*. *coli* O139 and O141 (data not shown). However it did increase the permeability of these strains to the NPN dye (Supplementary Figure 2). Taken together, these data indicate that MAC13243 is a promising lead molecule that can increase the efficacy of large-scaffold antibiotics, but that the molecular scaffold will need to be optimised if it is to be used as a potentiator.

### How does MAC13243 affect outer membrane permeability?

**Figure 5:**
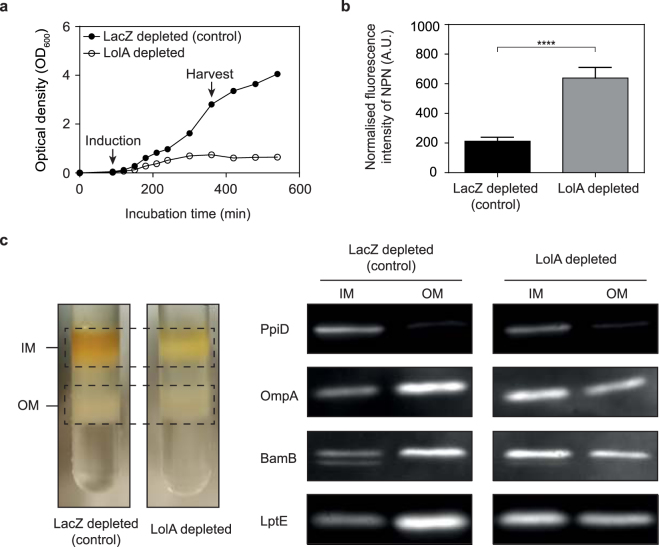
Partial depletion of LolA increases the permeability of the outer membrane. (**a**) CRISPRi-mediated knockdown of LolA or LacZ in *E*. *coli* MC4100. Expression of dCas9 together with the respective *lacZ* sgRNA (control) or *lolA* sgRNA was induced at t = 90 min with 200 ng mL^−1^ aTC and growth was monitored by measuring optical density (OD_600_). (**b**) An aliquot of cells was taken 4 h after induction and permeability was monitored by the NPN uptake assay. All data (mean +/− S.D.) are from four experiments. ****p < 0.0001 (unpaired t-test.). (**c**) Depletion of LolA levels by CRISPRi affects the trafficking of both lipoproteins and ß-barrel proteins to the outer membrane. Inner and outer membrane fractions were purified from both LacZ-depleted cells (control) and LolA-depleted cells using a sucrose gradient. The proteins from each fraction were then separated by SDS-PAGE, transferred to a nitrocellulose membrane and probed with anti-sera to an inner membrane protein (PpiD), an outer membrane protein (OmpA), and two outer membrane lipoproteins (BamB and LptE). Full-length blots are shown in Supplementary Figure 4.

MAC13243 is an inhibitor of LolA^21^, the periplasmic chaperone that traffics lipoproteins from the inner membrane to the outer membrane^13,34^. It was initially touted as a promising antibiotic lead because it targets an essential process, is effective against a collection of clinical isolates, and is not a substrate for efflux pumps^21^. The data presented here indicate that MAC13243 makes *E*. *coli* more permeable when used at sub-lethal concentrations. We therefore speculated that the permeable phenotype was caused by partial inhibition of LolA. To explore this possibility we mimicked partial inhibition by reducing the intra-cellular levels of LolA using the CRISPR interference (CRISPRi) technology^35^. When we induced expression of a single gRNA (sgRNA) specific for *lolA* together with the nuclease-deficient Cas9 (dCas9) we observed that cell growth was perturbed compared to control cells expressing an sgRNA for the non-essential gene *lacZ* (encoding ß–galactosidase) (Fig. 5a). This outcome was expected as *lolA* is essential for viability^36^ whereas *lacZ* is not^37^. When we monitored the permeability of LolA-depleted cells using the NPN assay, we observed that they were more permeable than LacZ-depleted cells (Fig. 5b). We could not confirm that LolA levels were depleted, as we did not have antisera to LolA. But we were able to show that outer membrane lipoproteins and ß-barrel proteins were partially retained at the inner membrane (Fig. 5c). In the control, we observed that an inner membrane protein (PpiD) was enriched in the inner membrane fraction, whereas an outer membrane protein (OmpA), and two lipoproteins (BamB, LptE) were enriched in the outer membrane fraction. However in LolA-depleted cells the outer membrane proteins were enriched in the inner membrane, which is consistent with our hypothesis that LolA levels were partially depleted by CRISPRi. The genetic reconstruction therefore demonstrates the principle that partial depletion of LolA is sufficient to make *E*. *coli* more permeable.

## Discussion

Antibiotic resistance is a major public health threat^38–40^. And although new antibiotics are urgently needed the antibiotic discovery pipelines are virtually empty, particularly for gram-negative bacteria^41–43^. Given this situation, it has been widely acknowledged that we should find better ways to use the antibiotics that we already have at our disposal^40,44–46^. With this philosophy in mind we set out to identify lead molecules that could make gram-negative bacteria more permeable, and more susceptible to large-scaffold antibiotics. Large-scaffold antibiotics are valuable community resources that were originally discovered as natural products, and decades of synthetic tailoring has created generations of molecules with improved characteristics^41^. These antibiotics are already in the public domain, but they are not used to treat gram-negative infections because they cannot efficiently cross the outer membrane^8,10,47–50^. We suggest that they represent an untapped community resource that could be repurposed to treat gram-negative infections, if there were approaches to increase the permeability of the outer membrane.

Our study identified MAC13243, which has previously been shown to be an inhibitor of the essential periplasmic chaperone LolA^21^. The previous work had shown that MAC13243 had antibacterial activity against gram-negative bacteria, and the authors suggested that it was a promising lead molecule. We too observed that MAC13243 had antibacterial activity (MIC ranging from 8 to 256 mg mL^−1^, depending on whether the cells were grown in LB or M9 minimal media). However a subsequent study noted that MAC13243 degrades in aqueous solution, casting doubt over its usefulness^51^.

**Figure 6:**
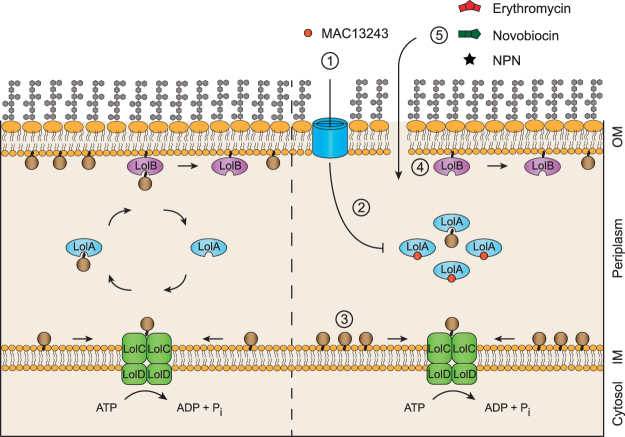
A model depicting how MAC13243 could affect the permeability of the outer membrane in *E*. *coli*. Lipoprotein precursors are synthesised on cytosolic ribosomes, then trafficked to the periplasm through either the Sec or Tat translocons. In the periplasm the N-terminal cysteine residue is acylated and then cleaved in successive reactions by Lgt, LspA and Lnt (not shown). Left panel, the mature lipoprotein is bound by the ABC transporter complex LolCDE then released to the periplasmic chaperone LolA. The LolA-lipoprotein complex is trafficked to the outer membrane where it binds to the LolB receptor and transfers the lipoprotein cargo. LolB then inserts the lipoprotein into the outer membrane. Note that some lipoproteins have a Lol avoidance signal and they are retained in the inner membrane. See^13^ for more details. Right panel, a sub-lethal concentration of MAC13243 (step 1) results in partial inhibition of LolA (step 2). This results in the partial retention of outer membrane lipoproteins at the inner membrane (step 3). Some of these mis-targeted lipoproteins are directly involved in outer membrane biogenesis, such as LolB (insertion of lipoproteins^13^), BamB (insertion of ß-barrel proteins^1^) and LptE (insertion of LPS molecules^52^). Thus partial depletion of LolA can directly affect the biogenesis of the key components of the outer membrane, which weakens the membrane and results in increased permeability (step 4). The increased permeability in cells treated with MAC13243 can be exploited to increase the uptake of NPN and large-scaffold antibiotics (step 5).

In this study we show for the first time, that sub-inhibitory concentrations of MAC13243 can be used to make *E*. *coli* more permeable. In our experiments we observed that *E*. *coli* cells were more permeable to the fluorescent dye NPN, as well as large-scaffold antibiotics from four different antibiotic classes when treated with sub-inhibitory concentrations of MAC13243. The fact that cells were more permeable to NPN than colistin-treated cells suggests that MAC13243 is effective at inducing a permeable phenotype. A molecular reason for the permeable phenotype is speculated on in Fig. 6. We reason that a sub-lethal concentration of MAC13243 results in partial inhibition of the LolA chaperone. Since LolA traffics proteins from the inner to the outer membrane, partial inhibition will result in retention of lipoproteins at the inner membrane. *E*. *coli* encodes around 90 different lipoproteins, many of which have no known function^13^. However three outer membrane lipoproteins are directly involved in outer membrane biogenesis and are essential for cell viability: LolB inserts lipoproteins into the outer membrane^13^, BamD inserts ß-barrel proteins into the outer membrane^1^ and LptE inserts LPS molecules into the outer membrane^52^. Thus partial inhibition of LolA can simultaneously affect the function of three proteins that are essential for maintaining the integrity of the outer membrane. Indeed, when we depleted the intracellular levels of LolA using CRISPRi, we observed that LptE was partially retained at the inner membrane, and that cells were more permeable to NPN. We were unable to determine if LolA-depletion was sufficient to cause susceptibility to large-scaffold antibiotics, since the CRISPRi system requires two plasmids with different antibiotic selection markers. Nevertheless the experiment indicated that LolA-depletion was sufficient to increase the permeability of *E*. *coli*.

**Figure 7:**
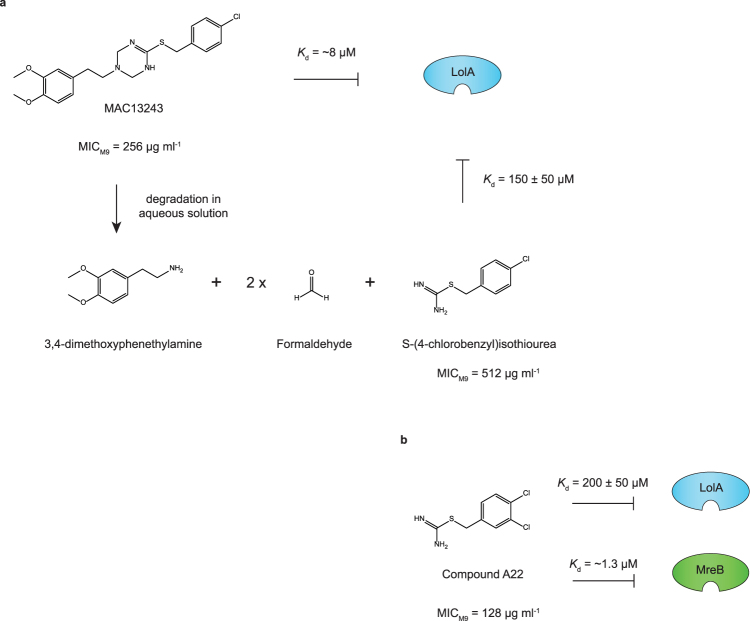
MAC13243 is degraded in solution. (**a**) MAC13243 is hydrolysed into one molecule of 3,4-dimethoxyphenethylamine, two molecules of formaldehyde and one molecule of *S-*(4-chlorobenzyl)isothiourea. At neutral pH the *t*_*1/*2_ is 13 h. Both MAC13243 and the degradation product *S-*(4-chlorobenzyl)isothiourea bind to LolA^21,51^. (**b**) An analogue of the degradation product, called A22 or *S-*(4-dichlorobenzyl)isothiourea, also binds LolA. Curiously this compound is a known inhibitor of the cytoskeletal protein MreB^51^. Figure adapted from^51^, with permission from the publisher.

Although the high throughput screen carried out in this study is unique, the molecule we identified (MAC13243) is similar to an antibiotic potentiator identified in a previous study^23^. In the aforementioned study the authors screened 30,000 compounds for their ability to potentiate the activity of novobiocin (also a large scaffold antibiotic). They identified A22 or *S-*(4-dichlorobenzyl)isothiourea, an inhibitor of the actin-like protein MreB. A22 is structurally similar to the thiourea moiety of MAC13243, which is liberated in aqueous solution as MAC13243 is hydrolysed^51^ (see *S-*(4-chlorobenzyl)isothiourea in Fig. 7). MAC13243, its degradation product *S-*(4-chlorobenzyl)isothiourea and A22 are of the same molecular class and it is therefore not surprising that they can all bind to LolA^21,51^ and cause the outer membrane to be more permeable to NPN (Fig. 3; Supplementary Figure 3). However it is surprising that two independent studies have identified essentially the same molecule as a potentiator of large-scaffold antibiotics, since there are literally hundreds of target proteins in *E*. *coli*. For example, *E*. *coli* is more susceptible to vancomycin when one of 60 different proteins is inactivated^12^.

Can MAC13243 be used as a potentiator of large-scaffold antibiotics? Whilst we observed that a sub-inhibitory concentration of MAC13243 worked synergistically with large-scaffold antibiotics like novobiocin and erythromycin in lab strains of *E*. *coli*, it did not work synergistically with larger scaffolds such as rifampicin and vancomycin. Furthermore it did not work synergistically with any of the tested antibiotics in clinical isolates (although we did observe increased permeability). Thus we suggest that MAC13243 will need to be chemically modified if it is to be used clinically as a potentiator of large-scaffold antibiotics. However it may have a number of immediate uses in biotechnology, such as improving the uptake of large labelling dyes or precursor compounds used for the production of complex chemicals in cell factories.

## Methods

### *Bacterial strains*, *chemicals and media*

The *E*. *coli* strain MC4100 (*F* ^*−*^, *[araD139]*_*B/r*_, *Δ(argF-lac)169*, *λ* ^*−*^, *e14-*, *flhD5301*, *Δ(fruK-yeiR)725(fruA25)*, *relA1*, *rpsL150(strR)*, *rbsR22*, *Δ(fimB-fimE)632(::IS1)*, *deoC1))* was used for all experiments unless otherwise stated. The *E*. *coli* O139 and O141 strains were obtained from Klas Udekwu (Stockholm University, Sweden). The BW25113 Δ*waaG* and *lptD4213* strains were obtained from Göran Widmalm (Stockholm University, Sweden) and Daniel Kahne (Harvard Medical School, USA), respectively. Strains were either grown in LB broth (Amresco, Stockholm) or M9 minimal medium containing 1x M9 minimal salts (BD Difco, Stockholm, Sweden), 0.4% D-glucose (VWR Chemicals, Stockholm, Sweden), 2 mM MgSO_4_ (Sigma Aldrich, Stockholm, Sweden) and 0.1 mM CaCl_2_ (Sigma Aldrich, Stockholm, Sweden). Antibiotics and other chemical compounds were purchased from the following manufacturers: Vancomycin, rifampicin, erythromycin, novobiocin and 1-(N-phenylamino)naphthalene (NPN) from Sigma Aldrich (Stockholm, Sweden); MAC13243 from MedChem Express (Stockholm, Sweden); 5-Floxuridine and Streptozotocin from Cayman Chemical (Michigan, USA); Carbadox from Alfa Aesar (Karlsruhe, Germany) and anhydrotetracycline (aTc) from VWR (Stockholm, Sweden).

### The High-Throughput Screen

The first part of the primary screen consisted of 17,500 compounds from a diverse set of small molecules (ChemBridge) screened at a final concentration of 12.5 µM. The second part consisted of 10,500 compounds from the CBCS primary screening set (Chemical Biology Consortium Sweden) screened at a final concentration of 10 µM. The compounds were Echo® spotted (Labcyte) directly into 96-well *Nunclon*™Δ surface plates (Thermo Fisher Scientific, Stockholm, Sweden). A colony of *E*. *coli* MC4100 was inoculated into LB broth and incubated overnight at 37 °C with shaking at 200 rpm. The overnight culture was diluted to OD_600_ = 0.01 and supplemented with 150 μg mL^−1^ vancomycin (100 µg mL^−1^ for the first 17,500 compounds). A 200 μL aliquot of diluted bacteria was then added to each well of the pre-spotted 96-well plates. The plates were incubated for 5 h at 37 °C without shaking and the OD_600_ were recorded with the Synergy H4 plate reader (BioTek). The growth control consisted of 200 μL diluted bacteria with 200 nl of DMSO. The sterility control consisted of 200 μL sterile growth media.

### Validation experiments

Selected small molecules were further analysed in dose-response experiments (with and without vancomycin). Briefly, diluted bacteria were prepared as described in the previous section. A 200 μL aliquot of diluted bacteria was either supplemented with or without 150 μg mL^−1^ vancomycin and added to a 96-well *Nunclon*™Δ surface plate and then mixed with two-fold serial dilutions of each small molecule at concentrations ranging from 0 - 20 μM. The plates were incubated for 5 h at 37 °C without shaking and the OD_600_ was recorded.

### Minimal Inhibitory Concentration (MIC) determination

The MIC of each antibiotic and small molecule was determined prior to the outer membrane integrity assays. A single colony of *E*. *coli* MC4100 was inoculated into either 5 mL LB broth or M9 minimal medium and incubated overnight at 37 °C and 200 rpm. The overnight culture was diluted with sterile growth medium to an OD_600_ = 0.0005. 198 μL of diluted cells per well were added to a 96-well *Nunclon*™Δ surface plate and mixed with 2 μL of two-fold serial dilutions of antibiotic or small molecule with a final concentration ranging from 0 to 2048 μg mL^−1^. After an 18 h incubation at 37 °C without shaking, the samples were transferred to a 96-well Costar plate (VWR, Stockholm, Sweden) and the OD_600_ was recorded in a *SpectraMax M2e* Microplate Reader (Molecular Devices, CA, USA). The growth control consisted of 198 μL diluted culture and 2 μL of the corresponding solvent. The sterility control consisted of 200 μL sterile growth medium and served as a background control. The MIC was defined as bacterial growth that was reduced by more than 90% compared to the growth control.

### Outer membrane integrity assays

The permeability of the outer membrane was analysed by using the NPN uptake assay as previously described^29^. Briefly, a colony of *E*. *coli* MC4100 was inoculated into 5 mL of M9 media and incubated overnight at 37 °C with shaking at 200 rpm. The overnight culture was diluted with sterile M9 medium to an OD_600_ = 0.1 and incubated in a 96-well *Nunclon*™Δ surface plate without shaking at 37 °C until the culture reached an OD_600_ = 0.5. The cells were harvested by centrifugation (15,000 x *g* for 2 min), washed twice with assay buffer (5 mM HEPES, 5 mM glucose, pH 7.2) and resuspended in assay buffer to a final OD_600_ = 1. Then, 100 μL of washed cells and 100 μL of assay buffer containing 20 μM NPN were mixed together and added to a 96-well optical-bottom black plate (Thermo Fisher Scientific, Stockholm, Sweden). Either 2 μL of a chemical compound, or the corresponding solvent, was added to each well and fluorescence was immediately monitored in a SpectraMax *Gemini EM* microplate reader (Molecular devices, CA, USA) at an excitation wavelength of 350 nm and emission wavelength of 420 nm for 10 min at 30 sec intervals. For each time point, the NPN uptake was calculated using equation (1) where F_obs_ is the observed fluorescence at a given chemical compound concentration, F_control_ is the fluorescence of NPN with *E*. *coli* cells in the presence of the corresponding solvent and F_B_ is the fluorescence of NPN in the absence of *E*. *coli* cells. Data collected over a 10 minutes was averaged.

1 $$NPN\,uptake=({F}_{obs}-{F}_{B})-({F}_{control}-{F}_{B})$$

### Susceptibility testing with large-scaffold antibiotics

A single colony of *E*. *coli* MC4100 was inoculated into 5 mL of LB broth and incubated overnight at 37 °C with shaking at 200 rpm. The overnight culture was diluted with sterile LB medium to an OD_600_ = 0.0005. A 196 μL aliquot of diluted cells was added to each well of a 96-well *Nunclon*™Δ surface plate and mixed with 2 μL of MAC13243 (final concentration of 10 μM) and/or 2 μL of either vancomycin (f.c. 128 μg mL^−1^), rifampicin (f.c. 8 μg mL^−1^), erythromycin (f.c. 128 μg mL^−1^) or novobiocin (f.c. 32 μg mL^−1^). The growth control contained 198 μL of diluted bacteria and 2 μL of DMSO. The sterility control contained 200 μL sterile growth medium and served as a background control. After an 18 h incubation at 37 °C without shaking, the OD_600_ was recorded as mentioned previously.

### Checkerboard dilution assay

Checkerboard dilution assays were performed to investigate if a given combination of MAC13243 and antibiotic worked synergistically. The protocol was adapted from^23^. Briefly, an overnight culture of *E*. *coli* MC4100 was diluted with sterile M9 medium to an OD_600_ = 0.0005. A 196 μL aliquot of diluted culture was added to each well of a 96-well *Nunclon*™Δ surface plate and mixed with either 2 μL of MAC13243 and/or 2 μL of antibiotic with concentrations ranging from 0 to 1024 μg mL^−1^. After an 18 h incubation at 37 °C without shaking, the OD_600_ was measured as described previously. The growth control consisted of 196 μL of diluted culture, 2 μL of DMSO and 2 μL of either H_2_O or EtOH. To evaluate if any given combination was synergistic, equation (2) was used to calculate the FIC (fractional inhibitory concentration) index where FIC_A_ is the MIC of drug A in combination with drug B divided by the MIC of drug A alone and FIC_B_ is the MIC of drug B in combination with drug A divided by the MIC of drug B alone. The combinations were deemed synergistic (FICI ≤ 0.5), additive (FICI > 0.5 to 1), indifferent (FICI > 1 to < 2) or antagonistic (FICI ≥ 2), as defined by community guidelines^33^.

2 $$FIC\,index=FI{C}_{A}+FI{C}_{B}=\frac{(MI{C}_{AB})}{MI{C}_{A}}+\frac{(MI{C}_{BA})}{MI{C}_{B}}$$

### CRISPRi

The sgRNA-expressing plasmids were constructed according to^53^. In short, a protospacer adjacent motif (PAM) sequence (5′-NGG-′3) and adjacent 20 nucleotides were selected on the non-template strand for *lacZ* and *lolA* (as close to the start codon as possible). Each sgRNA construct was transformed together with the dCas9-expression vector into *E*. *coli* MC4100 and selected on LB agar supplemented with 34 μg mL^−1^ chloramphenicol and 25 μg mL^−1^ kanamycin. A colony of each transformed strain was inoculated into 5 mL of LB medium containing the appropriate antibiotics and grown overnight at 37 °C with shaking at 200 rpm. The cultures were then diluted to OD_600 = _0.01, supplemented with fresh antibiotics and 5 mL of diluted cultures were added into a 24-well plate (GE Healthcare, Uppsala, Sweden). Cells were incubated at 37 °C with shaking at 200 rpm and expression of dCas9 and the corresponding sgRNA (sgRNA-LacZ or sgRNA-LolA) was induced with 200 ng mL^−1^ aTc at OD_600_ = 0.1.

### Membrane fractionation and western blot analysis

A single colony of *E*. *coli* MC4100 transformed with the dCas9-expression vector and the corresponding sgRNA (sgRNA-LacZ or sgRNA-LolA) was inoculated into 20 ml LB medium (containing the appropriate antibiotics) and incubated overnight at 37 °C with shaking at 200 rpm. The overnight cultures were diluted with sterile LB to an OD_600 = _0.01 in a final volume of 1 L. Incubation was continued under the same conditions for 2 h and expression of dCas9 and the corresponding sgRNA was induced with 200 ng mL^−1^ aTc. After an additional 5 h incubation under the same conditions, cells were harvested from 2 L of culture, by centrifugation for 20 min at 5000 × *g* at 4 °C. Harvested cells were resuspended in 1 × PBS and subsequently broken by passing three times through an Emulsiflex-C3 (Avestin, Mannheim, Germany). Unbroken cells were pelleted by centrifugation for 20 min at 8000 × *g* at 4 °C. The membrane fraction was pelleted from the supernatant by ultracentrifugation for 1 h at 270,000 × *g* at 4 °C. Membrane fractions were resuspended in 1 ml PBS, placed on top of a three-step sucrose gradient (0.77 M, 1.44 M and 2.02 M sucrose) and separated by ultracentrifugation for 16 h at 230,000 × *g* at 4 °C as described previously^54^. The inner and outer membrane fractions (IM + OM) were collected, then proteins were separated by 12% SDS-PAGE and blotted onto a nitrocellulose membrane using a semi-dry blotting device (Bio-Rad, Stockholm, Sweden). The nitrocellulose membranes were decorated with antisera, and detection was carried out using the ECL system (Thermo Scientific, Stockholm, Sweden) and a LAS-1000 CCD camera (Fujifilm).

## Electronic supplementary material

Supplementary information
