# Supplementary material for: Increasing the permeability of Escherichia coli using MAC13243
Source: Sci Rep. 2017 Dec 15;7:17629. doi: 10.1038/s41598-017-17772-6 (PMC5732295; doi:10.1038/s41598-017-17772-6)
Supplement: Supplementary file 1 — Supplementary information [file 41598_2017_17772_MOESM1_ESM.pdf]

Supplementary Information

Increasing the permeability of *Escherichia coli* using MAC13243

Claudio Muheim[a], Hansjörg Götzke[a], Anna U. Eriksson[b], Stina Lindberg[b],

Ida Lauritsen[c], Morten N. Nørholm[c] and Daniel O. Daley[a,*]

[a]Department of Biochemistry and Biophysics

Stockholm University, Stockholm, Sweden

[b]Chemical Biology Consortium Sweden,

Laboratories for Chemical Biology, Umeå, Sweden.

[c]Novo Nordisk Foundation Center for Biosustainability,

Technical University of Denmark, Copenhagen, Denmark.

* Address correspondence to: ddaley@dbb.su.se; +46 8 162910

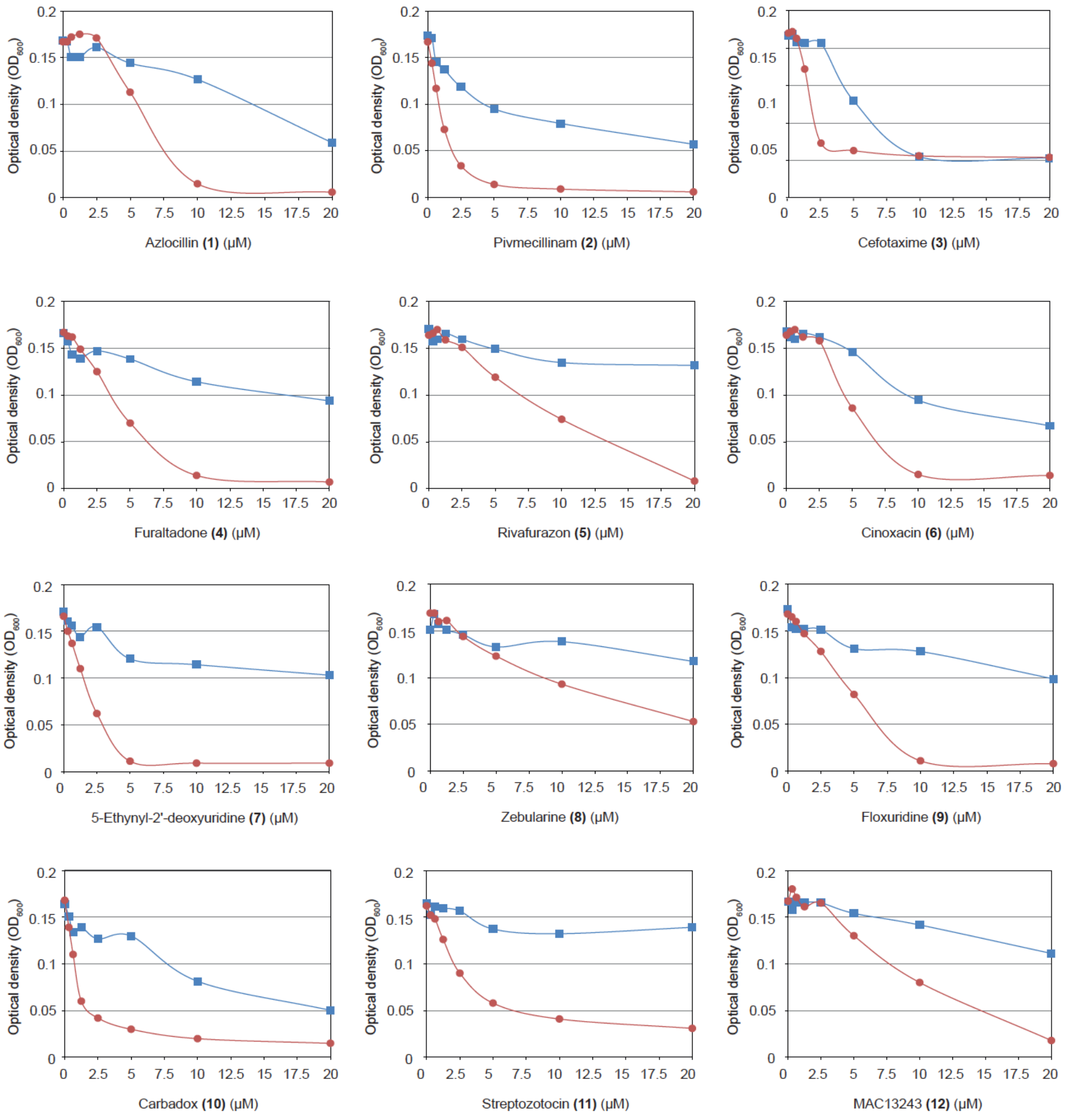

Supplementary Figure 1. Hits from the small molecule screen that inhibited cell growth in a vancomycin-dependent and dose-dependent manner. Blue curves illustrate cell growth (OD_{600}) in the presence of different concentrations of small molecules whereas red curves illustrate cell growth in the presence of different concentrations of small molecules and 150 µg mL^{-1} vancomycin.

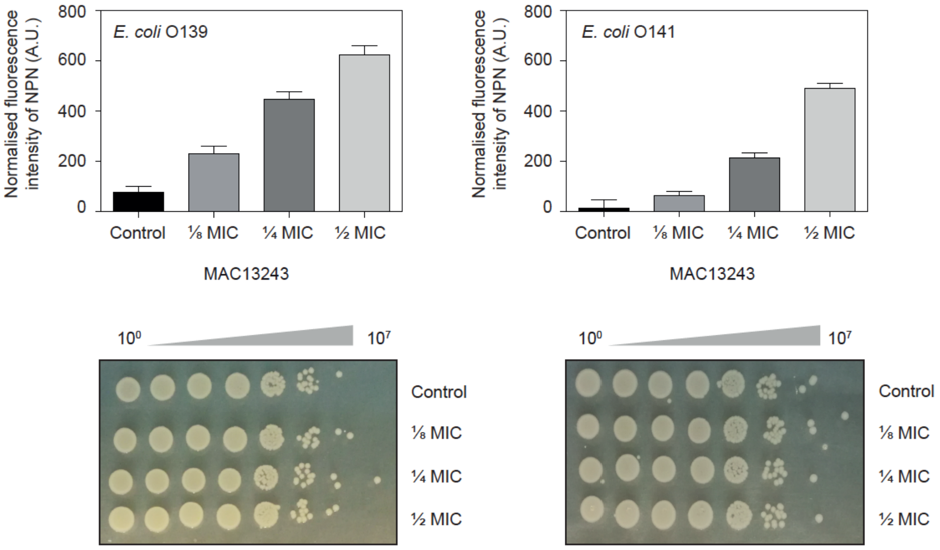

Supplementary Figure 2. A sub-lethal concentration of MAC13243 makes the outer membrane of *E. coli* O139 and O141 more permeable. Cells were grown in M9 media then exposed to different concentrations of MAC13234 (MIC = 256 µg mL^{-1}) and NPN uptake was monitored (top panels). The increase in fluorescence was deemed to be due to increased permeability of the outer membrane, not cell lysis, since the amount of MAC13243 used did not affect cell viability (bottom panels). In these experiments cell aliquots were harvested after the NPN uptake assays, 10-fold serially diluted and spotted on LB agar.

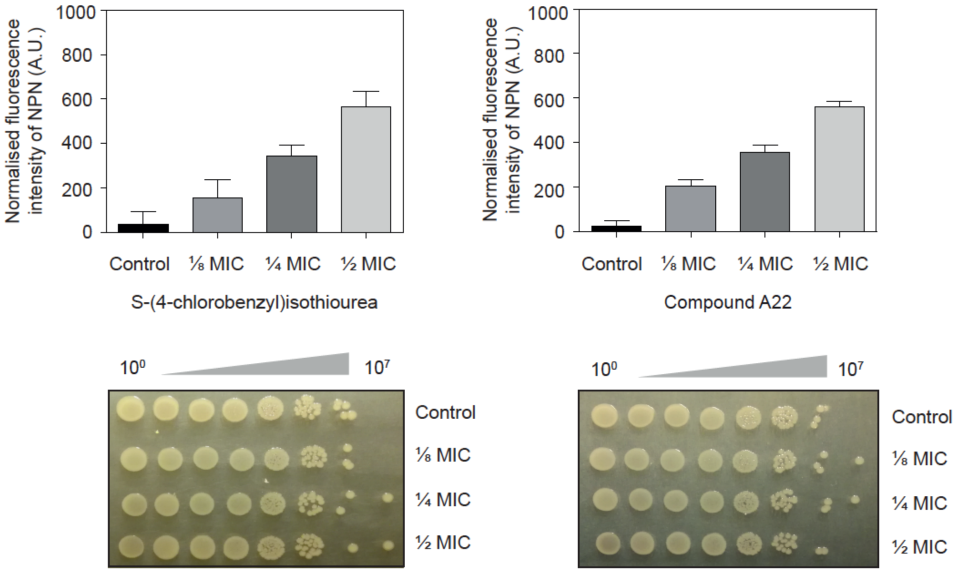

Supplementary Figure 3. A sub-lethal concentration of *S*-(4-chlorobenzyl)isothiourea or *S*-(4-dichlorobenzyl)isothiourea (A22) makes the outer membrane of *E. coli* MC4100 more permeable. Cells were grown in M9 media then exposed to different concentrations of the compounds and NPN uptake was monitored (top panels). The MIC of *S*-(4-chlorobenzyl)isothiourea was 512 µg mL^{-1}, and A22 was 128 µg mL^{-1}. The increase in fluorescence was deemed to be due to increased permeability of the outer membrane, not cell lysis, since the amount of compound used did not affect cell viability (bottom panels). In these experiments cell aliquots were harvested after the NPN uptake assays, 10-fold serially diluted and spotted on LB agar. All data (mean ± SD) are from four experiments.

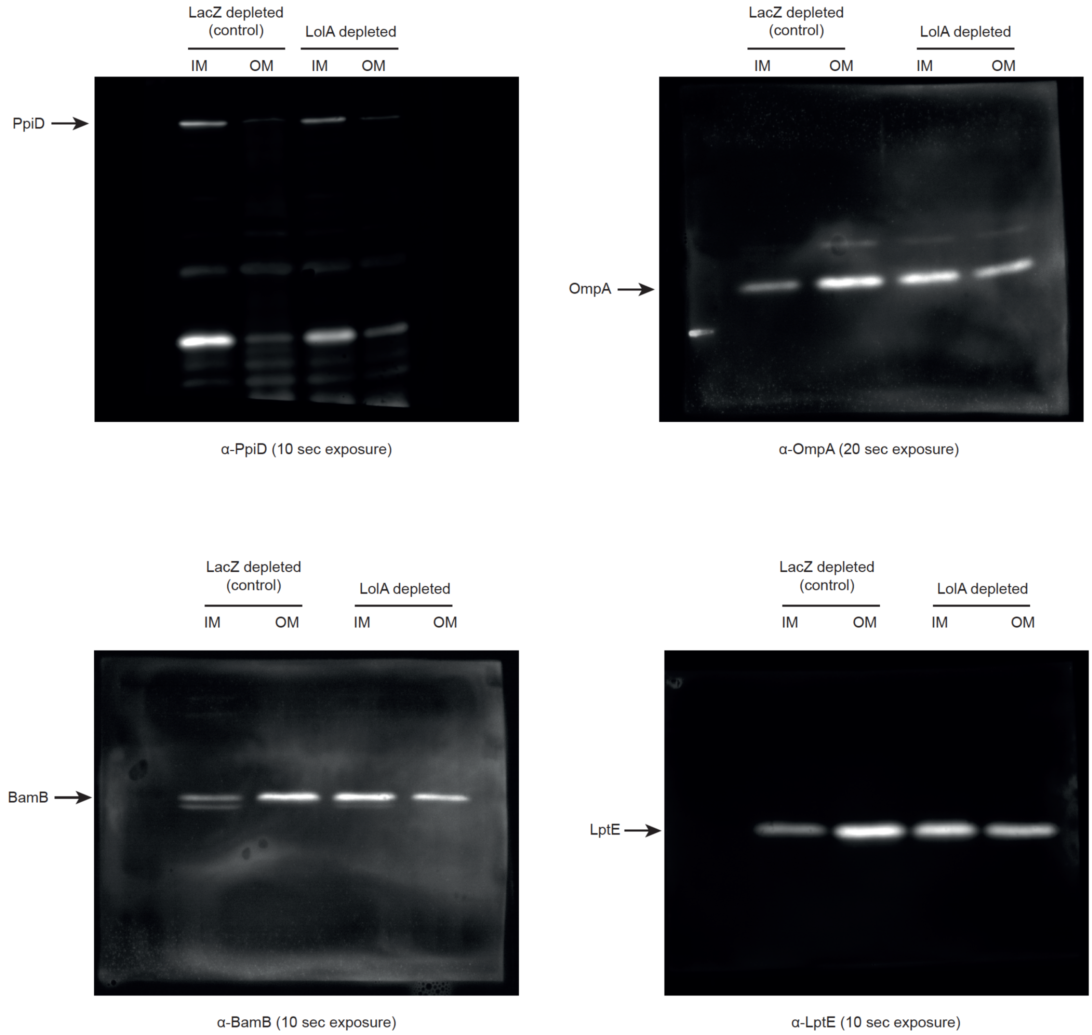

Supplementary Figure 4. Full-length blots of the experiment shown in Figure 5c of the manuscript.
